# The Mediation Effect of Hyperarousal Symptoms on the Relationship Between Childhood Physical Abuse and Suicidal Ideation of Patients With PTSD

**DOI:** 10.3389/fpsyt.2021.613735

**Published:** 2021-03-26

**Authors:** Aeran Kwon, Hyun Seo Lee, Seung-Hwan Lee

**Affiliations:** ^1^Department of Social Welfare and Counseling, Chodang University, Muan, South Korea; ^2^Clinical Emotion and Cognition Research Laboratory, Inje University, Goyang, South Korea; ^3^Department of Psychiatry, Inje University, Ilsan-Paik Hospital, Goyang, South Korea

**Keywords:** PTSD, suicide, childhood physical abuse, depression, hyperarousal

## Abstract

**Objective:** This study examined the relationship of childhood physical abuse, posttraumatic stress disorder (PTSD), depression, and suicide in patients with PTSD through path analysis.

**Materials and Methods:** A total of 114 patients with PTSD (36 men and 78 women) were recruited and completed psychological assessments including the Childhood Trauma Questionnaire, the scale for suicidal ideation, the clinician-administered PTSD scale for the *Diagnostic and Statistical Manual of Mental Disorders*, Fifth Edition, the PTSD checklist, and the Hospital Anxiety and Depression Scale. Structural equation modeling was used to evaluate the results. We developed a model including childhood physical abuse experience as the causal variable, suicidal ideation as a result variable, and PTSD and depression as mediation variables. PTSD symptoms were divided into four clusters [intrusion, avoidance, negative cognition and mood, and altered arousal and reactivity (hyperarousal)] to determine predictive power for suicide.

**Results:** PTSD symptoms fully mediated the relationship between childhood physical abuse and suicidal ideation. Furthermore, PTSD symptoms fully mediated the relationship between childhood physical abuse and depression. Among the PTSD symptoms, hyperarousal was the only symptom cluster that mediated the relationship between childhood physical abuse and suicidal ideation. The symptom clusters of negative cognition and mood as well as hyperarousal mediated the relationship between childhood physical abuse and depression.

**Conclusions:** This study presents a link between childhood physical abuse and current symptoms in patients with PTSD, and highlights specific PTSD symptom clusters (i.e., hyperarousal, negative cognition and mood) that may increase the risk for psychopathology later in life.

## Introduction

There are high rates of suicidal behavior among individuals diagnosed with posttraumatic stress disorder (PTSD) after being exposed to trauma (1, 2). According to several existing studies, the suicide attempt rates of patients with PTSD is between 24 and 40% (3–7). In patients with chronic PTSD, 56.4% experience suicidal behaviors, including suicidal ideation and suicide attempts (8).

Complex trauma in response to repeated and perpetuating experiences of interpersonal trauma usually shows more severe symptoms than simple trauma related to a single event, such as a car accident, natural disaster, or robbery (9–12). Childhood physical abuse is a leading example of complex trauma (13–15), and it has been reported that the influence of childhood physical abuse on suicidal behavior is serious (1, 16–18). In a study of 6,642 Canadians, a group that has experienced childhood physical abuse had a five-fold higher suicidal ideation when the group that did not experience such trauma (19). According to a national epidemiological survey of 43,093 individuals, the group that had experienced childhood physical abuse experienced higher levels of substance abuse disorders, ADHD, PTSD, and mood disorders, with higher rates of suicidal attempts compared to the group that had not experienced childhood physical abuse (17). Furthermore, PTSD diagnosis is one of the strongest predictors of recent and lifetime suicide attempts in adults who have experienced childhood abuse (20–22).

The number and severity of PTSD symptoms have been reported as key predictors of suicidal behavior in patients (5, 8, 23). According to a 15-year study that tracked the relationship between childhood trauma, PTSD occurrence, and suicide attempts with 1,698 adolescents, participants with a PTSD diagnosis had a significantly higher risk of suicide attempts compared to those exposed to trauma but not diagnosed with PTSD, and those not exposed to trauma (24).

Studies have examined the relationship between specific PTSD symptoms and suicidal behaviors. Most of these studies have utilized the Diagnostic and Statistical Manual of Mental Disorders, Fourth Edition (DSM-IV) criteria (25) dividing the symptoms into three clusters of reexperience, avoidance/numbing, and hyperarousal, to confirm their relationships with suicide. Some studies involving firefighters and soldiers reported that re-experiencing symptoms had a positive correlations with suicidal behavior (4, 6, 26, 27). Hyperarousal symptoms had a positive correlation with the risk of suicide in a community sample (6, 26, 28, 29). While there have been studies dividing PTSD symptoms into four clusters of intrusion, avoidance, negative cognition and mood, and altered arousal and reactivity based on the Diagnostic and Statistical Manual of Mental Disorders, Fifth Edition (DSM-5) criteria (28, 30–32), there are a limited number of studies related to suicidal behavior. In addition, studies based on the DSM-5 criteria do not control for demographic information and only measured the frequency of experiences of suicidal ideation and suicide attempts.

The main criticism of previous studies on PTSD and suicidality is that they overlook depression (23). Depression may mediate the relationship between PTSD and suicidal behaviors (33, 34), and the co-occurrence of PTSD and depression may exacerbate the effects of PTSD, leading to suicidal behavior (34).

**Figure 1:**
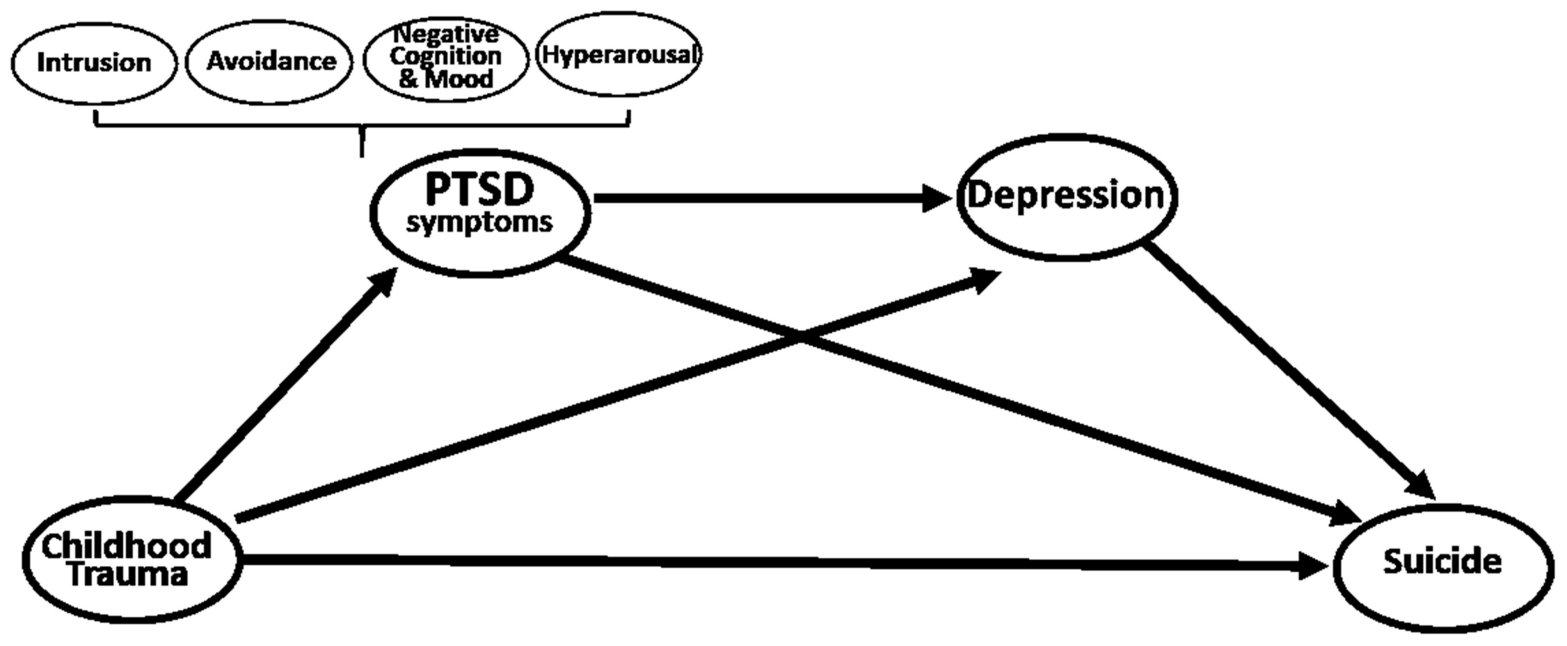
Hypothetical model: Multiple mediation model for effect of childhood trauma on suicide *via* PTSD symptoms and depression.

Our study examined the relationship of childhood physical abuse, PTSD, and depression and suicide in patients with PTSD through path analysis. We developed a model for patients with PTSD that included childhood physical abuse, PTSD symptoms (including four PTSD symptom clusters), depression, and suicide, and tested its validity (Figure 1). Confirming the relationship between the variables influencing suicidal behavior in patients with PTSD will present important implications in preventing suicide in patients with PTSD.

## Materials and Methods

### Participants

Patients with PTSD were recruited from the Psychiatry Department of the Inje University Ilsan Paik Hospital in Goyang, Korea. The diagnosis of PTSD made by a psychiatrist was based on the *DSM-5* full criteria. We excluded participants with a history or suffering from any psychiatric and/or neurological illness other than PTSD, such as schizophrenia, bipolar disorder, anorexia nervosa, intellectual disability, epilepsy, etc. A total of 146 patients with PTSD were registered for the study. Data sets from 32 individuals were excluded from the study [including 9 individuals who did not attempt or complete the psychological scale, 10 individuals who had high scores for denial on the Childhood Trauma Questionnaire (CTQ), and 13 individuals with severe brain damage]. Ultimately, data from 114 patients with PTSD were included in the analysis. The patients with PTSD included in the analysis were composed of 36 men (31.60%) and 78 women (68.40%), with an average age of 44.36 years (*SD* = 12.77) and an average education of 12.78 years (*SD* = 3.21). Each participant signed an informed consent form approved by the Institutional Review Board at Inje University Ilsan Paik Hospital before participating (IRB No. 2015-07-025).

### Psychological Measures

#### Childhood Trauma Questionnaire

The Korean validated version of the CTQ was used to evaluate childhood physical abuse (35). It is known as a useful self-report questionnaire in eliciting retrospective reports of childhood trauma (36). It consists of five subscales of various childhood trauma, including emotional, physical, and sexual abuse, and emotional and physical neglect as well as another scale for detecting minimization and denial. The CTQ consists of 28 items and is assessed with a 5-point Likert scale ranging from 1 (“never true”) to 5 (“very often true”). The coefficient alpha of the K-CTQ was 0.79 in this study, and the coefficient alphas of the 5 subscales were 0.89 (emotional abuse), 0.85 (sexual abuse), 0.90 (physical abuse), 0.61 (emotional neglect), and 0.95 (physical neglect).

#### Scale for Suicidal Ideation (SSI)

SSI a self-reported questionnaire for measuring suicidality (37). This study utilized the Korean validated version of the SSI (38). The scale consists of 19 items and is assessed using a 3-point Likert scale ranging from 0 to 2. The coefficient alpha of this scale was 0.91.

#### Clinician-Administered PTSD Scale for DSM-5 (CAPS-5)

CAPS-5 is a structured diagnostic interview that corresponds with the DSM-5 diagnosis for PTSD, administered by a psychiatrist (39). It consists of 30 items and assesses information about the frequency and severity of PTSD symptoms. This was assessed by standardizing and simplifying the conversion of symptom frequency and intensity ratings into dichotomous scores (“Yes” or “No”) and symptom severity (from 0 [“Absent”] to 4 [“Extreme/incapacitating”]). The coefficient alpha of the CAPS-5 severity score was 0.80.

#### Posttraumatic Stress Disorder Checklist-5 (PCL-5)

The PCL-50 is a self-reported questionnaire for measuring PTSD symptoms based on the DSM-5 and consists of a total of 20 questions (40). This study utilized the Korean validated version of the PCL-5 (41). The PCL-5 helps in the screening and diagnostic evaluation of PTSD and is used for the purpose of observing changes in PTSD symptoms. The results are measured using a Likert scale, ranging from 0 (“not at all”) to 4 (“extremely”), measuring the degree of pain one has experienced within the last month or since the previous stressful event. In addition, this study divided PTSD symptoms into four clusters based on the DSM-5. The four symptom clusters (intrusion, avoidance, negative cognition and mood, and altered arousal and reactivity) were individually included in the hypothetical model. In the text, altered arousal and reactivity are referred to as hyperarousal for convenience. The coefficient alpha of the PCL-5 score was 0.93 in this study.

#### Hospital Anxiety and Depression Scale (HADS)

HADS was used to evaluate symptoms of depression and anxiety (42). This study utilized the Korean validated version of HADS (43). It is a self-reported questionnaire with 7 items for describing anxiety and 7 items for describing depression. It is assessed with a 4-point Likert scale ranging from 0 (“no problems”) to 3 (“maximum distress”). The coefficient alphas for the subscales of HADS were 0.92 (anxiety) and 0.83 (depression) in this study.

### Statistical Analysis

Partial correlation analysis with bootstrapping at a 1,000-sampling rate was performed to examine the correlations among the variables. Sex, age, years of education, and type of trauma were controlled.

To utilize the maximum likelihood estimation method in the structured equation model, this study confirmed the skewness and kurtosis of the variables. Skewness over 3 and kurtosis over 7 are considered to be a moderately non-normal distribution (44).

Exploratory factor analysis was performed to establish the latent variables. The scale was parceled using the Factor-Parceling Approach based on the size of the factor loads (45, 46). SSI and PCL-5 were each grouped into a total of three factors, with six to seven items per parcel. As for the childhood physical abuse and the HADS-depression scale, all individual items were used without any parceling.

To analyze the convergent validity and discriminant validity of the latent variables, this study conducted a confirmatory factor analysis and utilized the maximum likelihood estimation method. Values of 0.50 or higher for standardized factor load are reported to indicate satisfactory convergent validity (47), in accordance with the criteria of discriminant validity, which states that a correlation of 0.80 or higher between latent variables indicates the measurement of the same concept. To test reliability, Cronbach's α values were calculated.

To evaluate the fit of the measurement model, this study calculated χ^2^, χ^2^/df, comparative fit index (CFI), normed fit index (NFI), incremental fit index (IFI), Tucker Lewis Index (TLI), goodness of fit index (GFI), adjusted goodness of fit index (AGFI), root mean squared error of approximation (RMSEA), root mean-square residual (RMR), and standardized root mean squared residual (SRMR). A good model is defined as when χ^2^/df is <3, the CFI, NFI, IFI, TLI, GFI, and AGFI are >0.90, and the RMSEA and SRMR are <0.08 (44).

To test whether PTSD symptoms and depression mediate the relationship between childhood physical abuse and suicidal ideation, this study used the serial multiple mediation model as the structural model. Furthermore, the four structural models including the 4 PTSD symptom clusters were set to specifically investigate the effect of PTSD symptoms on suicidal ideation in patients with PTSD. The fit of the structural model was calculated, and the validity of the structural model was confirmed by using the maximum likelihood estimation to analyze the validity of the parameters. Next, this study identified the total, direct, and indirect effects of the model. To evaluate the significance of the indirect effects, this study used a bootstrapping method to generate 1,000 samples (48). Phantom variables were used to confirm the size and significance of the specific indirect effects (49).

This study examined the hypothetical model using AMOS 20 for testing the structural equation model (SPSS, Inc., Chicago, IL, USA). Statistical analyses were performed using SPSS 18 (SPSS, Inc., Chicago, IL, USA).

## Results

### Descriptive Statistics

Among 114 patients with PTSD, 34 experienced trauma from interpersonal relationships such as sexual abuse or domestic abuse (29.6%), and 80 (70.2%) experienced non-relationship trauma, such as automobile accidents, falls, and kidnappings. The frequency, mean, standard deviation, skewness, and kurtosis of the variables are shown in Table 1.

**Table 1:** Demographics and clinical characteristics of patients with PTSD (*N* = 114).

	**Mean or (*N*)**	**SD or (%)**	**Skewness**	**Kurtosis**
**Demographics**				
Sex				
Male	(36)	(31.60)		
Female	(78)	(68.40)		
Age [yrs]	44.36	12.77	−0.33	−0.87
Education [yrs]	12.78	3.21	−0.76	0.35
Life traumatic event				
Interpersonal trauma	(34)	(29.80)		
Non-interpersonal trauma	(80)	(70.20)		
**Clinical characteristics**				
Suicidal ideation	10.54	7.68	0.44	−0.79
Childhood physical abuse	10.32	6.06	1.09	0.00
PTSD symptoms	37.79	17.42	−0.03	−0.66
Depression	5.08	1.58	0.33	0.02

Significant positive correlations were identified between suicidal ideation and childhood physical abuse (*r* = 0.198, *p* < 0.05), suicidal ideation and PTSD symptoms (*r* = 0.440, *p* < 0.001), suicidal ideation and depression (*r* = 0.362, *p* < 0.001), childhood physical abuse, and PTSD symptoms (*r* = 0.263, *p* < 0.01), and PTSD symptoms and depression (*r* = 0.534, *p* < 0.001). In contrast, the correlations between childhood physical abuse and depression was not statistically significant.

### Tests of the Measurement Model

This study found that the standardized factor loading value was higher than 0.50, indicating no issues with convergent validity in the measurement model (47). The correlations between latent variables ranged between 0.184 and 0.643, meeting the criteria of 0.80 or below, indicating that the measurement model has discriminant validity. The Cronbach's α values of the latent variables ranged from 0.717 to 0.924, which indicates reliability. The fit of the measurement model was found to be acceptable, with χ^2^ = 97.822 (*p* = 0.144), IFI = 0.987, TLI = 0.984, CFI = 0.987, RMSEA = 0.038, and SRMR = 0.043 (44).

### Test of the Structural Model

The results indicated that the fit of the structural model including four major variables (i.e., childhood physical abuse, PTSD symptoms, depression, and suicidal ideation) was acceptable, with χ^2^/df = 144.034/127 = 1.134, IFI = 0.985, TLI = 0.979, CFI = 0.984, and RMSEA = 0.034.

**Figure 2:**
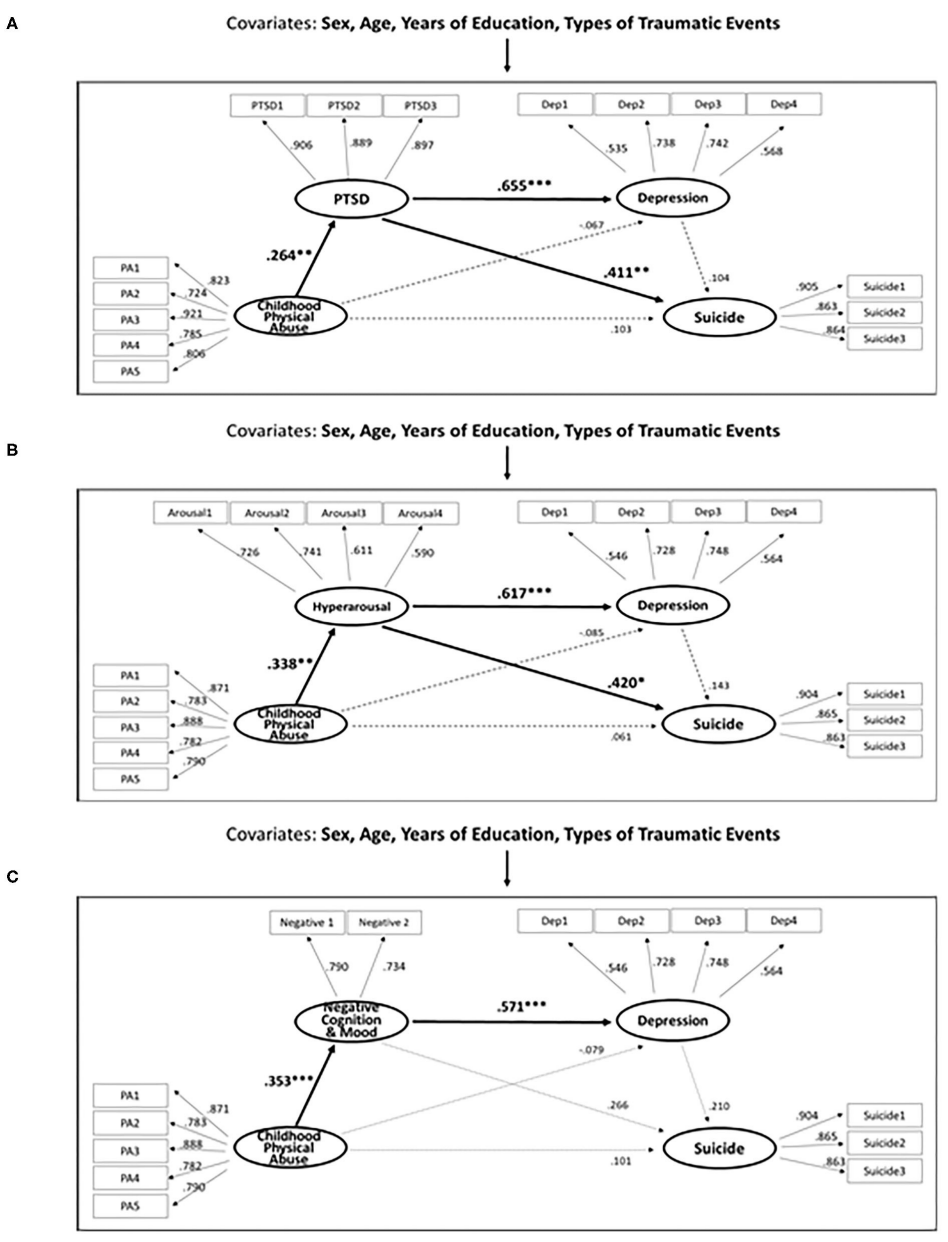
**(A)** Structural equation model of the effects of childhood physical abuse, PTSD and depression on suicide in patients with PTSD (*N* = 114). **(B,C)** Structural equation model of the effects of childhood physical abuse, specific PTSD symptom cluster and depression on suicide in patients with PTSD (*N* = 114).

The results of the effect test indicated that PTSD symptoms fully mediate the relationship between childhood physical abuse and suicidal ideation in patients with PTSD (Figure 2A). More specifically, the total effect of childhood physical abuse on suicidal ideation was significant (β = 0.222, *p* = 0.040). The direct effect of childhood physical abuse on suicidal ideation was not significant (β = 0.103, *p* = 0.304). The indirect effect of PTSD symptoms between childhood physical abuse and suicidal ideation was significant (β = 0.119, *p* = 0.039). The specific indirect effect of PTSD symptoms between childhood physical abuse and suicidal ideation was significant (*B* = 0.009, *p* = 0.010) (Table 2).

**Table 2:** Standardized total, direct, indirect, and specific indirect effects of structural model in patients with PTSD (*N* = 114).

**Path**	**Total**	***p***	**Direct**	***p***	**Indirect**	***p***
	**effect**		**effect**		**effect**	
Childhood PA → Suicide	0.222	0.040	0.103	0.304	0.119	**0.039**
Childhood PA → PTSD	0.264	0.016	0.264	**0.016**		
PTSD → Depression	0.655	0.002	0.655	**0.002**		
Depression → Suicide	0.104	0.453	0.104	0.453		
PTSD → Suicide	0.479	0.002	0.411	**0.010**	0.068	0.453
Childhood PA → Depression	0.106	0.371	−0.067	0.517	0.173	**0.016**
Sex → Suicide	−0.034	0.726	0.026	0.724	−0.060	0.202
Age → Suicide	−0.094	0.315	0.028	0.777	−0.121	0.013
Education → Suicide	−0.171	0.105	−0.059	0.545	−0.112	0.034
Types of trauma → Suicide	−0.243	0.022	−0.199	0.038	−0.044	0.371
Sex → PTSD	−0.103	0.256	−0.013	0.261		
Age → PTSD	−0.261	0.012	−0.261	0.033		
Education → PTSD	−0.209	0.033	−0.209	0.012		
Types of trauma → PTSD	−0.105	0.261	−0.105	0.256		
Sex → Depression	−0.169	0.132	−0.102	0.724	−0.068	0.256
Age → Depression	−0.143	0.230	0.029	0.777	−0.171	0.012
Education → Depression	−0.254	0.035	−0.118	0.545	−0.137	0.034
Types of trauma → Depression	−0.012	0.922	0.057	0.038	−0.069	0.261
**Specific indirect effect**			**Effect**	***p***	**Boot LLCI**	**Boot ULCI**
Childhood PA → PTSD → Depression Suicide			0.002	0.454	−0.005	0.012
Childhood PA → PTSD → Suicide			0.009	**0.010**	0.000	0.035
Childhood PA → Depression → Suicide			−0.001	0.708	−0.007	0.004
Childhood PA → PTSD → Depression			0.015	**0.002**	0.001	0.056

Controlled for sex, age, years of education, and type of trauma as covariates.

PA, physical abuse; LLCI, ULCI: lower and upper limits within the 95% confidence interval of the mediation effect.

*The bold values mean related variables for significant mediation effect*.

Furthermore, PTSD symptoms fully mediated the relationship between childhood physical abuse and depression (Figure 2A). More specifically, the total effect of childhood physical abuse on depression was not significant (β = 0.106, *p* = 0.371). The direct effect of childhood physical abuse on depression was not significant (β = −0.067, *p* = 0.517). The indirect effect of PTSD symptoms between childhood physical abuse and depression was significant (β = 0.173, *p* = 0.016). The specific indirect effect of PTSD symptoms between childhood physical abuse and depression was significant (*B* = 0.015, *p* = 0.002) (Table 2). The results show that PTSD symptoms act as important mediators in the relationship where childhood physical abuse, possibly leading to the development of depression or suicide among patients with PTSD.

However, the specific indirect effect of PTSD symptoms and depression between childhood physical abuse and suicidal ideation (*B* = 0.002, *p* = 0.454), and the specific indirect effect of depression between childhood physical abuse and suicidal ideation (*B* = −0.001, *p* = 0.708) were not statistically significant.

### Test of the Structural Model for Specific PTSD Symptom Clusters

The fit of the four structural models for specific PTSD symptom clusters were all acceptable as follows: χ^2^/df <3, CFI, NFI, IFI, TLI, GFI, and AGFI are more than 0.90, and RMSEA and SRMR are <0.08 (Supplementary Table 1) (44).

The results of the effect test indicated that the hyperarousal cluster was the only cluster that fully mediated the relationship between childhood physical abuse and suicidal ideation (Figure 2B). More specifically, the direct effect of childhood physical abuse on hyperarousal (β = 0.338, *p* = 0.002) and the direct effect of hyperarousal on suicidal ideation (β = 0.420, *p* = 0.038) were significant. The indirect effect of childhood physical abuse on suicidal ideation through hyperarousal (*B* = 0.042, *p* = 0.041) was significant. Furthermore, the hyperarousal cluster fully mediated the relationship between childhood physical abuse and depression (Figure 2B). In other words, the direct effect of hyperarousal on depression (β = 0.617, *p* = 0.002) and the indirect effect of childhood physical abuse on depression through hyperarousal (*B* = 0.068, *p* = 0.003) were significant. Finally, the negative alterations in cognition and mood fully mediated the relationship between childhood physical abuse and depression (Figure 2C). In other words, the direct effect of childhood physical abuse on negative alterations in cognition and mood (β = 0.353, *p* = 0.004), the direct effect of negative alterations in cognition and mood on depression (β = 0.571, *p* = 0.002), and the indirect effect of childhood physical abuse on depression through negative alterations in cognition and mood (*B* = 0.063, *p* = 0.001) were significant. The direct effects, indirect effects, and specific indirect effects using bootstrapping of the four structural models are presented in Supplementary Table 2.

## Discussion

The purpose of this study was to explore the relationship between childhood physical abuse, PTSD symptoms, depression, and suicidal ideation in patients with PTSD. The results indicated that PTSD symptoms fully mediated the relationship between childhood physical abuse and suicidal ideation. Furthermore, PTSD symptoms fully mediated the relationship between childhood physical abuse and depression. Among the symptom clusters, hyperarousal fully mediated the relationship between childhood physical abuse and suicidal ideation. The negative alterations in cognition and mood and hyperarousal fully mediated the relationship between childhood physical abuse and depression.

PTSD symptoms fully mediated the relationship between childhood physical abuse and suicidal ideation. These results are similar with previous studies which pinpointed PTSD symptom as one of the major factors related to suicide among PTSD patients (26, 34, 50, 51). This study is also in line with existing studies showing that people who have experienced childhood physical abuse were more likely to be diagnosed with PTSD (52–56). PTSD symptoms can be understood as the internal state of fear, experienced through childhood physical abuse, that persists in current lives (57). PTSD symptoms was reported to appear when a traumatic experience in childhood have not been fully integrated or assimilated into memory, and individuals have difficulty processing the experience as a past event that can no longer bring harm (58). In addition, people who have experienced childhood physical abuse are vulnerable to different types of trauma in adulthood (59), which can lead to more serious and a higher frequency of suicide behaviors (60). In summary, our current results provide a link between childhood physical abuse and later life psychopathology in patients with PTSD.

PTSD symptoms fully mediate the relationship between childhood physical abuse and depression in patients with PTSD. Previous studies have shown that PTSD is a major predictor of the co-occurrence of depression (61–68). Our results support that patients with a primary diagnosis of PTSD had identified depressive symptoms as being one of the most frequent co-occurrence.

Among the PTSD symptom clusters, only hyperarousal mediated the relationship between childhood physical abuse and suicidal ideation. Hyperarousal refers to symptoms of anxiety, hypervigilance, difficulty concentrating, and irritability (69) and includes deficits in emotion regulation, impulsivity, and aggressive and self-destructive behavior (70, 71). The association between hyperarousal and suicidal behavior may reflect the tendency of severely traumatized patients with PTSD to crave a way out of their emotional pain, and do so through ending their lives (72). Studies reporting an association between anxiety sensitivity and suicidal behavior support this interpretation (73–75). Anxiety sensitivity is referred to as the fear of arousal sensation, and bodily sensations associated with autonomic arousal as a sign of imminent personal harm. Trauma survivors suffering from persistent hyperarousal showed extreme levels of fear over losing cognitive control (76). Patients with PTSD are thought to perceive suicidal behavior as a strategy to avoid an agonizing internal state (76). In short, suicidal ideation in patients with PTSD might be related to one of the PTSD patients' responses to try and extinguish intense pain in the midst of post-traumatic hyperarousal. Therefore, our findings can help identify those who are at a higher risk for suicidal ideation and provide early therapeutic interventions.

The hyperarousal PTSD symptom cluster fully mediated the relationship between childhood physical abuse and depression. As mentioned above, those with serious hyperarousal symptoms demonstrate violent, aggressive, and self-destructive behaviors, damaging themselves and others (77). Furthermore, they have difficulties in social relationships, as they are often blamed or shunned by people around them and find adaptation difficult (78), which can ultimately increase depression. This is in line with existing studies that aggressive and impulsive behaviors possibly leading to the development of depression (78, 79).

The negative alterations in cognition and mood fully mediated the relationship between childhood physical abuse and depression. A higher number of adults and adolescents who have experienced childhood abuse were currently suffering from depressive disorders compared to those who did not have the same experience (80). A longitudinal study also found that people who have experienced childhood physical abuse were more likely to have later depressive disorder (81). The depression of those who suffered from childhood physical abuse has been shown to be related to helplessness, guilt, and loss of attachment experienced through childhood physical abuse (82, 83). Our result is consistent with prior findings in the sense that childhood physical abuse indirectly predicted depression through mediating of the negative alterations in cognition and mood cluster. Because this cluster emphasizes negative cognition and mood related to trauma, it significantly overlaps with depression (84). The negative alterations in cognition and mood cluster could be used to identify those with PTSD symptoms, which later develop into co-occurrence of depression.

There are some limitations to this study. First, this study examined mediation effects based on cross-sectional data of patients with PTSD. Therefore, it is difficult to find causality in the relationships between the variables. Although the CTQ is regarded as a reliable tool for measuring childhood trauma in many studies (85–90), additional longitudinal studies would be helpful to support the findings from this study. Second, although the minimum sample size was 114, given an effect size (*f* ^2^) of 0.15, significance criterion of 0.05, power at 0.80, and 9 for the number of predictor variables (91), a replication study is needed to generalize the current findings. Third, although the type of trauma was controlled as a covariate, a further investigation separating PTSD patients with relational trauma from those with non-relational trauma is needed.

In conclusion, this study is meaningful as it was to our knowledge the first attempt to examine the mediation effect of PTSD symptoms in the relationship between childhood physical abuse and suicidal ideation in patients with PTSD. Although the relationship between PTSD and suicidality has been previously established, the current findings offer progress of this knowledge, as it sheds light on the importance of specific PTSD symptoms (i.e., hyperarousal, negative cognition and mood). It also provides a link between childhood physical abuse and current symptoms, and highlight hyperarousal symptoms that may increase the risk of suicidal ideation in later life. Therefore, treatment that focuses on alleviating hyperarousal symptoms would be helpful to prevent suicidal ideation in patients with PTSD who have experienced childhood physical abuse.

## Data Availability Statement

The original contributions presented in the study are included in the article/Supplementary Material, further inquiries can be directed to the corresponding author/s.

## Ethics Statement

The studies involving human participants were reviewed and approved by Institutional Review Board at Inje University Ilsan Paik Hospital (IRB No. 2015-07-025). The patients/participants provided their written informed consent to participate in this study.

## Author Contributions

AK performed study conception, design, methodology, formal analysis, and writing-original draft. HSL performed data collection and writing-editing. S-HL performed funding acquisition, investigation, review, and supervision. All authors discussed the results and commented on the manuscript at all stages, and approved the final manuscript.

## Conflict of Interest

The authors declare that the research was conducted in the absence of any commercial or financial relationships that could be construed as a potential conflict of interest.
