# Supplementary material for: The Mediation Effect of Hyperarousal Symptoms on the Relationship Between Childhood Physical Abuse and Suicidal Ideation of Patients With PTSD
Source: Front Psychiatry. 2021 Mar 26;12:613735. doi: 10.3389/fpsyt.2021.613735 (PMC8032896; doi:10.3389/fpsyt.2021.613735)
Supplement: Supplementary file 1 [file Data_Sheet_1.docx]

Supplementary Materials

1. **Supplementary Tables**

| **Supplementary Table 1.** Model fit indices of the four structural models for specific PTSD symptom cluster in patients with PTSD (*N*=114). | | | | | | | | | | | |  |
| --- | --- | --- | --- | --- | --- | --- | --- | --- | --- | --- | --- | --- |
| **Model** | ***χ^2^*** | ***df*** | ***p*** | ***χ^2^*** | **IFI** | **GFI** | **AGFI** | **TLI** | **CFI** | **RMSEA** | **SRMR** |  |
|  |  |  |  |  |  |  |  |  |  |  |  |  |
| Model A | 169.674 | 128 | .008 | 1.326 | .957 | .872 | .811 | .939 | .955 | .054 | .049 |  |
| Model B | 168.256 | 128 | .010 | 1.314 | .958 | .871 | .809 | .942 | .956 | .053 | .048 |  |
| Model C | 137.552 | 111 | .044 | 1.239 | .972 | .888 | .827 | .959 | .970 | .046 | .047 |  |
| Model D | 173.855 | 146 | .058 | 1.191 | .971 | .874 | .819 | .961 | .970 | .041 | .052 |  |

*Note.* Model A included intrusion cluster as a mediator; Model B included avoidance cluster as a mediator; Model C included altered cognitions and mood cluster as a mediator; Model D included altered arousal and reactivity cluster as a mediator.

**Supplementary Table 2.** Standardized total, direct, indirect effects, and specific indirect effects of four structural models for specific PTSD symptom cluster (*N*=114)

**① Effects of Model A (Intrusion cluster as a mediator)**

| **Path** | | **Total**  **Effect** | | ***p*** | | **Direct**  **Effect** | | ***p*** | | | **Indirect**  **Effect** | | | ***p*** | |
| --- | --- | --- | --- | --- | --- | --- | --- | --- | --- | --- | --- | --- | --- | --- | --- |
| Childhood PA 🡪 Suicide | | .218 | | .033 | | .126 | | .231 | | | .092 | | | .080 | |
| Childhood PA 🡪 Intrusion | | .203 | | .059 | | .203 | | .059 | | |  | | |  | |
| Intrusion 🡪 Depression | | .420 | | .006 | | .420 | | .006 | | |  | | |  | |
| Depression 🡪 Suicide | | .189 | | .174 | | .189 | | .174 | | |  | | |  | |
| Intrusion 🡪 Suicide | | .420 | | .002 | | .340 | | .013 | | | .079 | | | .180 | |
| Childhood PA 🡪 Depression | | .120 | | .316 | | .035 | | .741 | | | .085 | | | .065 | |
|  | |  | |  | |  | |  | | |  | | |  | |
| **Specific indirect effect** | | | | | **Effect** | | | | ***p*** | | | **Boot LLCI** | | **Boot ULCI** | |
| Childhood PA | 🡪 Intrusion 🡪Depression | | 🡪 Suicide | | | | .005 | | | .211 | | | -.002 | .020 |  |
| Childhood PA | 🡪 Intrusion | | 🡪 Suicide | | | | .020 | | | .072 | | | -.001 | .053 |  |
| Childhood PA | 🡪 Depression | | 🡪 Suicide | | | | .002 | | | .083 | | | -.016 | .023 |  |
| Childhood PA | 🡪 Intrusion | | 🡪 Depression | | | | .027 | | | .064 | | | -.001 | .069 |  |

*Note.* Controlled for sex, age, years of education, and type of trauma as covariates

*Abbreviation.* PA: Physical Abuse; LLCI, ULCI: lower and upper limits within the 95% confidence interval of the mediation effect

**② Effects of Model B (Avoidance cluster as a mediator)**

| **Path** | | **Total**  **Effect** | | ***p*** | | **Direct**  **Effect** | | ***p*** | | | **Indirect**  **Effect** | | | ***p*** | |  |
| --- | --- | --- | --- | --- | --- | --- | --- | --- | --- | --- | --- | --- | --- | --- | --- | --- |
| Childhood PA 🡪 Suicide | | .221 | | .030 | | .176 | | .096 | | | .044 | | | .326 | |  |
| Childhood PA 🡪 Avoidance | | .087 | | .422 | | .087 | | .422 | | |  | | | .000 | |  |
| Avoidance 🡪 Depression | | .548 | | .002 | | .548 | | .002 | | |  | | | .000 | |  |
| Depression 🡪 Suicide | | .211 | | .197 | | .211 | | .197 | | |  | | | .000 | |  |
| Avoidance 🡪 Suicide | | .335 | | .003 | | .220 | | .119 | | | .116 | | | .197 | |  |
| Childhood PA 🡪 Depression | | .120 | | .306 | | .072 | | .499 | | | .048 | | | .422 | |  |
|  | |  | |  | |  | |  | | |  | | |  | |  |
| **Specific indirect effect** | | | | | **Effect** | | | | ***p*** | | | **Boot LLCI** | | **Boot ULCI** | |  |
| Childhood PA | 🡪Avoidance 🡪 Depression | | 🡪 Suicide | | | | .003 | | | .526 | | | -.006 | | .019 | |
| Childhood PA | 🡪 Avoidance | | 🡪 Suicide | | | | .005 | | | .609 | | | -.012 | | .029 | |
| Childhood PA | 🡪 Depression | | 🡪 Suicide | | | | .006 | | | .488 | | | -.010 | | .029 | |
| Childhood PA | 🡪 Avoidance | | 🡪 Depression | | | | .015 | | | .422 | | | -.023 | | .059 | |

*Note.* Controlled for sex, age, years of education, and type of trauma as covariates

*Abbreviation.* PA: Physical Abuse; LLCI, ULCI: lower and upper limits within the 95% confidence interval of the mediation effect

**③ Effects of Model C (Negative cognition and mood cluster as a mediator)**

| **Path** | | **Total**  **Effect** | ***p*** | | **Direct**  **Effect** | | | ***p*** | | **Indirect**  **Effect** | | | ***p*** | | |  |  |
| --- | --- | --- | --- | --- | --- | --- | --- | --- | --- | --- | --- | --- | --- | --- | --- | --- | --- |
| Childhood PA 🡪 Suicide | | .220 | .029 | | .101 | | | .432 | | .119 | | | .082 | | |  |  |
| Childhood PA 🡪 Negative c&m | | .353 | .003 | | .353 | | | .004 | |  | | |  | | |  |  |
| Negative c&m 🡪 Depression | | .571 | .002 | | .571 | | | .002 | |  | | |  | | |  |  |
| Depression 🡪 Suicide | | .210 | .172 | | .210 | | | .251 | |  | | |  | | |  |  |
| Negative c&m 🡪 Suicide | | .386 | .018 | | .266 | | | .136 | | .120 | | | .172 | | |  |  |
| Childhood PA 🡪 Depression | | .122 | .261 | | -.079 | | | .496 | | .201 | | | .003 | | |  |  |
|  | |  |  | |  | | |  | |  | | |  | | |  |  |
| **Specific indirect effect** | | | | | | **Effect** | | | ***p*** | | **Boot LLCI** | | | **Boot ULCI** | | | |
| Childhood PA | 🡪 Negative c&m🡪Depression | | | 🡪 Suicide | | | .012 | | .127 | | | -.004 | | | .051 | |  |
| Childhood PA | 🡪 Negative c&m | | | 🡪 Suicide | | | .028 | | .084 | | | -.007 | | | .086 | |  |
| Childhood PA | 🡪 Depression | | | 🡪 Suicide | | | -.005 | | .406 | | | -.042 | | | .011 | |  |
| Childhood PA | 🡪 Negative c&m | | | 🡪 Depression | | | **.063** | | **.001** | | | **-.024** | | | **.139** | |  |

*Note.* Controlled for sex, age, years of education, and type of trauma as covariates

*Abbreviation.* PA: Physical Abuse; Negative c&m: Negative cognition and mood; LLCI, ULCI: lower and upper limits within the 95% confidence interval of the mediation effect

**④ Effects of Model D (Hyperarousal cluster as a mediator)**

*Note.* Controlled for sex, age, years of education, and type of trauma as covariates

| **Path** | | **Total**  **Effect** | | ***p*** | | **Direct**  **Effect** | | ***p*** | | | **Indirect**  **Effect** | | | ***p*** | |  |
| --- | --- | --- | --- | --- | --- | --- | --- | --- | --- | --- | --- | --- | --- | --- | --- | --- |
| Childhood PA 🡪 Suicide | | .220 | | .040 | | .061 | | .616 | | | .160 | | | .036 | |  |
| Childhood PA 🡪 Hyperarousal | | .338 | | .002 | | .338 | | .002 | | |  | | |  | |  |
| Hyperarousal 🡪 Depression | | .617 | | .002 | | .617 | | .002 | | |  | | |  | |  |
| Depression 🡪 Suicide | | .143 | | .408 | | .143 | | .408 | | |  | | |  | |  |
| Hyperarousal 🡪 Suicide | | .508 | | .002 | | .420 | | .038 | | | .088 | | | .409 | |  |
| Childhood PA 🡪 Depression | | .123 | | .318 | | -.085 | | .529 | | | .209 | | | .003 | |  |
|  | |  | |  | |  | |  | | |  | | |  | |  |
| **Specific indirect effect** | | | | | **Effect** | | | | ***p*** | | | **Boot LLCI** | | **Boot ULCI** | |  |
| Childhood PA | 🡪Hyperarousal 🡪Depression | | 🡪 Suicide | | | | .009 | | | .407 | | | -.023 | | .034 | |
| Childhood PA | 🡪 Hyperarousal | | 🡪 Suicide | | | | **.042** | | | **.041** | | | **.002** | | **.113** | |
| Childhood PA | 🡪 Depression | | 🡪 Suicide | | | | -.004 | | | .753 | | | -.029 | | .118 | |
| Childhood PA | 🡪 Hyperarousal | | 🡪 Depression | | | | **.068** | | | **.003** | | | **.019** | | **.144** | |

*Abbreviation.* PA: Physical Abuse; LLCI, ULCI: lower and upper limits within the 95% confidence interval of the mediation effect
